# Controlling behavior, power relations within intimate relationships and intimate partner physical and sexual violence against women in Nigeria

**DOI:** 10.1186/1471-2458-11-511

**Published:** 2011-06-29

**Authors:** Diddy Antai

**Affiliations:** 1 Center for Global & Population Health, The Angels Trust Nigeria, Casablanca Street, Wuse 2, Abuja, Federal Capital Territory (FCT), Nigeria; 2 Karolinska Institute, Department of Public Health, Division of Social Medicine, 171 76 Stockholm, Sweden

## Abstract

**Background:**

Controlling behavior is more common and can be equally or more threatening than physical or sexual violence. This study sought to determine the role of husband/partner controlling behavior and power relations within intimate relationships in the lifetime risk of physical and sexual violence in Nigeria.

**Methods:**

This study used secondary data from a cross-sectional nationally-representative survey collected by face-to-face interviews from women aged 15 - 49 years in the 2008 Nigeria Demographic and Health Survey. Utilizing a stratified two-stage cluster sample design, data was collected frrm 19 216 eligible with the DHS domestic violence module, which is based on the Conflict Tactics Scale (CTS). Multivariate logistic regression analysis was used to determine the role of husband/partner controlling behavior in the risk of ever experiencing physical and sexual violence among 2877 women aged 15 - 49 years who were currently or formerly married or cohabiting with a male partner.

**Results:**

Women who reported controlling behavior by husband/partner had a higher likelihood of experiencing physical violence (RR = 3.04; 95% CI: 2.50 - 3.69), and women resident in rural areas and working in low status occupations had increased likelihood of experiencing physical IPV. Controlling behavior by husband/partner was associated with higher likelihood of experiencing physical violence (RR = 4.01; 95% CI: 2.54 - 6.34). In addition, women who justified wife beating and earned more than their husband/partner were at higher likelihood of experiencing physical and sexual violence. In contrast, women who had decision-making autonomy had lower likelihood of experiencing physical and sexual violence.

**Conclusion:**

Controlling behavior by husband/partner significantly increases the likelihood of physical and sexual IPV, thus acting as a precursor to violence. Findings emphasize the need to adopt a proactive integrated approach to controlling behavior and intimate partner violence within the society.

## Background

It is commonly accepted that control and power are underlying factors for sexual and other forms of violence by intimate partners [1,2]. Control in intimate partner relationships can be conceptualized as a problem of one partner (commonly the man) using threats and emotional abuse to maintain control over the other partner (commonly the women) [3,4]. Studies have shown that controlling behavior among men is significantly associated with higher likelihood of physical violence [5,6], and sexual violence [7,8], given that controlling behaviors reflect a power motive. Men who justify wife beating to control and discipline their wife are more physically aggressive than those who do not support such beliefs [9]. Few studies have however focused on controlling behavior as the crux of their research, more so in the sub-Saharan African context; most of the existing studies have mainly been carried out in North America [7], the United Kingdom [10], Asia [11], and the Middle East [12]. Focusing on the role of control in intimate relationships will increase our understanding of the etiology and consequences of male-to-female physical and sexual violence and is important in informing efforts towards prevention and reduction of IPV against women, given that controlling behavior is more common than physical or sexual violence, and can be equally or more threatening than physical or sexual violence [13,14]. Being controlled by an intimate partner and the use of emotional threats are highly injurious behaviors resulting in adverse effects on well-being [15], and warrants as much focus as other forms of violence, such as physical and sexual violence [4,14,16].

The approach in this study takes into consideration that control within the relationship has multiple forms and sources, and its manifestation may be influenced by personal attributes, institutional roles and cultural contexts [8]. It examines different dimensions of control within the Nigerian context in emphasizing some of the theoretical concepts offered for the causes of intimate partner violence. One of such theoretical explanations is the *feminist theory*, which posits that societal-level power imbalances within patriarchal societies create structural factors that work directly or/and indirectly to validate a male-dominated social order and family structure that often result in men exercising power and control over women in several ways, one of which is violence [2,17]. In this study, I use two variables to assess *feminist theory*; *i*) controlling behavior, which is used to assess male-dominated family structures and social order of patriarchal societies that encourage men to exercise control over women, as well as test the hypothesis that controlling behavior is a precursor to violence and is associated with increased likelihood of violence; and *ii*) women's justification of wife beating by a husband/partner, which is reflective of the cultural context in which the women reside, and traditional societal norms that legally permit a man to inflict physical punishment on his wife/partner, with the women generally accepting such violent acts against them. *Patriarchal perspectives *posit that violence may occur as a response to a man's feeling of powerlessness as well as of being threatened by a loss of control over an independent spouse [18]. Items which reflect women's independence from their spouse/partner and potential feelings of "powerlessness" of the male spouse/partner, in addition to the presence of violence and controlling behaviors, are considered as reflecting *patriarchal perspectives *in this study.

*Resource perspectives *on the other hand posit that it is the relative resources of male partners and women rather than social roles or norms that determine the balance of power within intimate relationships thereby increasing the risk of IPV [7]. A key element when examining control within relationships is the unequal social distribution of power between sexes as well as economic dependence i.e. gender inequality [8]. Individuals with less power are often the victims of control by those with more power. The dimensions of inequality within the relationship used in this study include spouses' relative earning, spouses' relative education, spouses' relative age, and type of marital union. Relationships of equally dependent partners that embrace egalitarian decision-making and an equal division of power within the family often report low levels of conflict, control, and violence [19,20]. Women who differ from the societal gender roles may be regarded as challenging their partner's masculinity as provider or breadwinner; these partners may resort to using control tactics to curtail such "deviant" behavior, which may result in violence [21]. For instance, women whose economic resources approached or exceeded their partners' resources were more likely to report experiencing control and violence acts [19]. Thus, in certain contexts the greater the equality (or less inequality) between partners, the higher the women's risk of exposure to violence as this threatens men's position of power [3,22]. Thus, this study uses variables reflecting relationship inequalities to assess the *resource perspectives*.

A variant of the resource perspective, *social exchange theory*, also posits that interpersonal dimensions of control and power can be expressed by decision-making autonomy, and the ability to engage in actions against a partner's wishes. Thus, greater power is held by the partner who maintains control over his/her partner's actions and decision-making within the relationship. Decision-making autonomy is used to assess *social exchange theory *in this study.

The aims of this study were to: *i*) assess the prevalence of controlling behavior, physical and sexual intimate partner violence against women; and *ii*) to assess the role of husband/partner controlling behavior in the risks of physical and sexual intimate partner violence against women.

I hypothesized that women who reported less power within their relationship would most likely be victims of controlling behavior and be at a increased risk of physical and sexual IPV.

## Methods

### Design and procedures

This study used data from the 2008 Nigeria Demographic and Health Survey (NDHS). This is a cross-sectional population-based survey that utilizes a stratified two-stage cluster sample design based on the list of enumeration areas developed from the 2006 Population Census sampling frame. A full report and detailed description of the sampling procedures are presented elsewhere [23]. All women (N = 33385) aged 15 - 49 years who were residents or visitors in the sampled households at the time of the survey were eligible for inclusion. However, the DHS domestic violence module, which is based on a modified and previously validated version of the Conflict Tactics Scale (CTS) [24], was used to obtain data on domestic violence from only one randomly chosen woman in each household (N = 19216 or 56% of eligible women). This was carried out in accordance with the World Health Organization's ethical and safety recommendations for research on domestic violence [25]. These recommendations aim at ensuring women's safety, maximizing disclosure of actual violence by providing adequate training and support to field workers, making sure informed consent is obtained, and guaranteeing the privacy of respondents.

### Measures

#### Outcome variable

IPV exposure is defined as any acts of physical, emotional and sexual abuse by a current or former partner whether cohabiting or not [26]. Using the CTS, two outcome variables of violence were created: physical and sexual violence. Physical violence referred to any exposure to one or several of the following acts against women by a current or former husband or partner ever: *i*) pushing, shaking or throwing something at her; *ii*) slapping her or twisting her arm; *iii*) punching or hitting her with something harmful; *iv*) kicking or dragging her; *v*) strangling or burning her; *vi*) threatening her with a weapon (e.g. gun or knife); and *vii*) attacking her with a weapon. Sexual violence referred to any exposure to one or several of the following acts against women by a current or former husband or partner ever: *i*) forced sexual intercourse; and *ii*) other sexual acts when undesired. Exposure to each of these types of violent acts were scored as 1 (any experience of violence ever) and 0 (no experience of violence ever). Reliability of the items, measured by Cronbach's alpha (α) was established prior to analyses (α = .90 for physical violence, and α = .83 for sexual violence).

### Exposure

*a) Controlling behavior*, the main exposure variable of interest, was assessed as a composite dichotomous "yes" or "no" variable made up of responses to six questions about whether present or former husband/partner had control issues i.e. if husband/partner: jealous if she talks with other men, accuses her of unfaithfulness, does not permit her to meet her friends, tries to limit her contact with family, insists on knowing where she is, and doesn't trust her with money. Women who responded "yes" to one or several of the control questions formed one group of the dichotomy, and the women that responded "no" to all the controlling attitude questions formed the other group of the dichotomy. Similar scales have been used in other studies [27,28]. Cronbach's alpha for controlling behavior was .90.

*b) *Other measures of relationship control included: *i) justifies wife beating*, a composite variable created from responses to five questions about whether the women would justify partner abuse of a woman for one of these reasons: if she goes out without telling him; if she neglects the children; if she argues with him; if she refuses to have sex with him; and if she burns the food. Responses were transformed into a single dichotomous "yes" or "no" variable. Women who responded "yes" to one or several of these attitude questions formed one group of the dichotomy, and women who responded "no" to all the attitude questions formed the other group of the dichotomy. Cronbach's alpha for justifies wife beating was .88; and *ii*) *autonomy in domestic decisions*, a composite variable assessed as a dichotomous "yes" or "no" variable created from responses to 5 questions about whether the women had the final say regarding: large household purchases; daily household purchases; visits to family or friends; own health; and deciding what to do with husband's money. Women whose response was either "respondent alone" or "respondent and husband/partner" to one or several of these questions formed one group of the dichotomy, while women who responded "respondent and other person in the household" and "no" to all these questions formed the other group of the dichotomy. Cronbach's alpha for autonomy in domestic decisions was .89.

*c) *Variables reflecting relationship inequalities included: *i*) *spouses' relative earning*; *ii*) *spouses' relative education*; *iii*) *spouses' relative age*. Each of these variables was categorized as "same", "less", and "more" in comparison to husband/partner; and *iv*) *type of union*, categorized as monogamy (i.e. no other wife) and polygamy (i.e. ≥ 1 other wife).

*d) *Demographic and socioeconomic variables as confounders included: *i*) *women's age*, grouped as ≤ 24, 25 - 34, ≥ 35 years; *ii*) *women's education *and *iii*) *partner's education*, categorized as no education, primary education, and secondary or higher education; *iv*) *women's occupation *and *v*) *partner's occupation*, categorized as professional, technical management; clerical, sales, skilled manual; agricultural self-employed, agricultural employee, household & domestic, unskilled manual; and not working (not working category was excluded in partner's occupation for having only 3 respondents); and *vi*) *place of residence*, categorized as urban and rural.

### Ethical considerations

This study is based on analysis of secondary data with all participant identifiers removed. The survey procedure and instruments used have received ethical approval from the National Ethics Committee in the Federal Ministry of Health of Nigeria and the Ethics Committee of the Opinion Research Corporation Macro international, Inc. (ORC Macro Inc, Calverton, MD; USA). Permission to use the DHS data in this study was obtained from ORC Macro Inc.

### Statistical analysis

Cross-tabulation was used to study the association between physical and sexual violence (outcome) and the key variables (exposures), using Pearson's chi-squared test (χ) to analyze significant differences, as well as variables indicating controlling behavior. Multivariate logistic regression analysis was used to examine the association between outcome and exposure variables, and results presented in the form of relative risk (RR) and 95% confidence intervals (CI). Covariates were entered and statistical significance using p-values was set at *p *< 0.05. Model 1 contained only husband/ partner's controlling behavior so as to show the gross effects of controlling behavior on the risk of physical and sexual violence before netting out the effects of other variables.

Model 2 added variables reflecting other measures of control in relationships (justifies wife beating and decision-making autonomy), and Model 3 further added variables reflecting possible relationship inequality (spouses' relative earning, spouses' relative education, spouses' relative age, and type of union). Model 4 included demographic and socioeconomic characteristics (women's age, women's education, women's occupation, partner's occupation, and place of residence). Missing data were excluded from the analyses. The analyses were performed using PASW Statistics version18.0 [29].

## Results

### Lifetime prevalence of controlling behavior, physical and sexual violence

The lifetime prevalence of controlling behavior was high with a significant proportion of women reporting exposure to at least one form of controlling behavior (63%) by their husband/male partner. The lifetime prevalence of physical violence was 15% and of sexual violence was 3%.

### Distribution of physical and sexual violence, controlling behavior and selected characteristics

**Figure 1:**
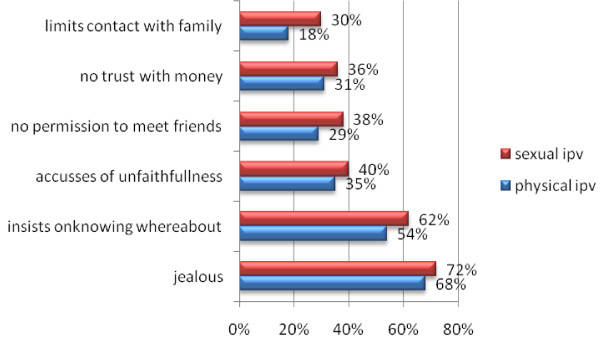
**Proportion of respondents who reported items used to measure controlling behavior by intimate partner violence**.

The proportion of respondents who reported items measuring controlling behavior are presented according to physical and sexual IPV in Figure 1. Women having a jealous spouse/partner most commonly reported physical (68%) and sexual (72%) IPV, while women whose spouse/partner limited them having contact with family were least likely to report physical (18%) and sexual (30%) IPV. Of the women who had been subjected to one or more controlling tactics by their husband/male partner (as indicated by the composite variable), 2256 (79%) had been exposed to physical violence (*p*< 0.000), and 563 (85%) had been exposed to sexual violence (*p*< 0.000). In addition, 1749 (61%; *p*< 0.000) and 439 (65%; *p*< 0.000) of the women who justified wife beating had been exposed to physical and sexual violence, respectively. Of the women who had decision-making autonomy, 2105 (73%; *p*< 0.000) had been exposed to physical violence and 478 (62%; *p*< 0.000) had been exposed to sexual violence. More women in monogamous relationships (n = 2056; 71%) reported being subjected to physical violence (*p*< 0.023) compared to those in polygamous relationships, and 290 (44%) women in monogamous relationships reported experiencing sexual violence (*p*< 0.000). More women with clerical/sales/services/skilled manual occupations (n = 1273; 44%), whose partner had secondary or higher education (n = 1307; 45%) and were agricultural self-employed/ agricultural employee/household & domestic/unskilled manual workers (n = 1321; 47%) had experienced physical violence, while more women who were clerical/sales/services/skilled manual workers (n = 305; 43%) had experienced sexual violence.

A higher proportion of the women reported experiencing physical violence by their husband/ partner who had secondary of higher education (n = 1285; 45%), and were agricultural self-employed/ agricultural employee/household & domestic/unskilled manual workers (n = 1294; 45%). Finally, more women resident in rural areas had experience physical violence (n = 2118; 74%) and sexual violence (n = 498; 75%) (Table 1).

**Table 1:** Distribution of lifetime prevalence of exposure to physical and sexual violence by controlling behaviours, relationship inequality, demographic and socioeconomic characteristics

	Physical violence		Sexual violence	
	
Characteristics	N = 2877 (%)	P-value	N = 663 (%)	P-value
*Husband/partner has controlling behavior (composite variable)*		0.000		0.000

No	621 (21)		100 (15)	

Yes	2256 (79)		563 (85)	

*Justifies wife beating*		0.000		0.000

No	1128 (39)		225 (34)	

Yes	1749 (61)		438 (66)	

*Decision-making autonomy*		0.000		0.000

No	772 (27)		185 (28)	

Yes	2105 (73)		478 (62)	

*Type of marriage*		0.023		0.465

Monogamy	2056 (71)		290 (44)	

Polygamy	821 (29)		373 (56)	

*Women's age*		0.000		0.415

≤ 24	572 (20)		168 (25)	

25 - 34	1263 (44)		279 (42)	

≥ 35	1042 (36)		216 (33)	

*Women's education*		0.000		0.000

No education	996 (35)		168 (25)	

Primary	925 (32)		279 (42)	

Secondary or higher	956 (33)		216 (33)	

*Women's occupation*		0.000		0.000

Profession/technical/management	95 (3)		25 (4)	

Clerical/sales/services/skilled manual	1273 (44)		293 (44)	

Agricultural self-employed/agricultural employee/ household & domestic/ unskilled manual	914 (32)		173 (26)	

Not working	595 (21)		172 (26)	

*Partner's education*		0.000		0.000

No education	813 (28)		199 (30)	

Primary	779 (27)		210 (32)	

Secondary or higher	1285 (45)		254 (38)	

*Partner's occupation*		0.001		0.149

Profession/technical/management	232 (8)		56 (8)	

Clerical/sales/services/skilled manual	1351 (47)		323 (49)	

Agricultural self-employed/agricultural employee/ household & domestic/ unskilled manual	1294 (45)		284 (43)	

*Place of residence*		0.000		0.010

Urban	759 (26)		165 (25)	

Rural	2118 (74)		498 (75)	

### Association between the risk of lifetime exposure to physical and sexual violence, controlling behavior, and selected characteristics

Controlling behavior of husband/partner was significantly associated with physical violence (RR = 2.77; 95% CI: 2.51 - 3.05) in the unadjusted analysis (Model 1); this association remained even more significant after adjusting for demographic and socioeconomic characteristics (RR = 3.04; 95% CI: 2.50 - 3.69) in Model 4. In the final model (Model 4), other measures of controlling behavior (justifies wife beating and decision-making autonomy), relationship inequalities (spouses' relative earnings), demographic and socioeconomic characteristics (women's occupation and place of residence) were significantly associated with physical violence after adjusting for possible confounding with these variables. Women who justified wife beating had a higher likelihood of experiencing physical abuse (RR = 1.66; 95% CI: 1.40 - 1.96) than women who did not justify wife beating, and women with decision-making autonomy had lower likelihood of experiencing physical abuse (RR = 0.71; 95% CI: 0.59 - 0.86) than women without decision-making autonomy. Women who earned more than their spouse/partner (RR = 1.69; 95% CI: 1.07 - 1.53), who were agricultural self-employed/agricultural employee/ household & domestic/ unskilled manual workers (RR = 3.00; 95% CI: 1.99 - 4.51), and those residing in rural areas (RR = 1.28; 95% CI: 1.06 - 1.56) had a higher likelihood of experiencing physical violence compared to women who earned as much as their husbands/partners, who were professional/technical/ management workers, and resided in urban areas, respectively (Table 2).

**Table 2:** Adjusted odds ratios with 95% confidence intervals for controlling behavior and risks of lifetime exposure to physical violence

	Model 1^a^	Model 2^b^	Model 3^c^	Model 4^d^
	
Characteristics	RR (95% CI)	RR (95% CI)	RR (95% CI)	RR (95% CI)
**Control in relationships**				

*Controlling behavior*				

No	1	1	1	1

Yes	2.77 (2.51 - 3.05)	2.51 (2.26 - 2.79)	2.87 (2.38 - 3.48)	3.04 (2.50 - 3.69)

*Justifying wife beating*				

No		1	1	1

Yes		1.55 (1.42 - 1.69)	1.57 (1.34 - 1.84)	1.66 (1.40 - 1.96)

*Decision-making autonomy*				

No		1	1	1

Yes		0.49 (0.44 - 0.54)	0.59 (0.49 - 0.70)	0.71 (0.59 - 0.86)

**Relationship inequality**				

*Spouses' relative earnings*				

Woman earns same			1	1

Woman earns less			0.89 (0.64 - 1.25)	1.09 (0.77 - 1.53)

Woman earns more			1.35 (0.86 - 2.10)	1.69 (1.07 - 1.53)

Woman's partner does not contribute			0.60 (0.36 - 0.98)	0.74 (0.44 - 1.22)

*Spouses' relative education*				

Woman has same			1	1

Woman has less			0.73 (0.28 - 1.89)	0.87 (0.33 - 2.31)

Woman has more			1.45 (1.22 - 1.73)	1.20 (0.91 - 1.58)

*Spouses' relative age*				

Woman same age			1	1

Woman younger			1.45 (0.69 - 3.08)	1.37 (0.64 - 2.93)

Woman older			1.05 (0.34 - 3.30)	0.97 (0.31 - 3.70)

*Type of union*				

Monogamy			1	1

Polygamy			1.02 (0.85 - 1.21)	1.09 (0.91 - 1.32)

**Demographic & socioeconomic characteristics**				

*Women's age*				

≤ 24				0.98 (0.77 - 1.23)

25 - 34				1.20 (1.00 - 1.43)

≥ 35				1

*Women's education*				

No education				0.73 (0.41 - 1.28)

Primary				1.08 (0.77 - 1.52)

Secondary or higher				1

*Women's occupation*				

Professional/technical/management				1

Clerical/sales/services/skilled manual				1.52 (0.33 - 2.31)

Agricultural self-employed/agricultural employee/ household & domestic/unskilled manual				3.00 (1.99 - 4.51)

Not working				1.73 (0.46 - 3.92)

*Partner's education*				

No education				0.64 (0.38 - 1.07)

Primary				1.00 (0.72 - 1.38)

Secondary or higher				1

*Partner's occupation*				

Professional/technical/management				1

Clerical/sales/services/skilled manual				1.00 (0.72 - 1.41)

Agricultural self-employed/agricultural employee/household & domestic/unskilled manual				0.98 (0.68 - 1.41)

*Place of residence*				

Urban				1

Rural				1.28 (1.06 - 1.56)

-2 Log likelihood	15428.90	13600.19	4414.89	4261.09

^a ^Controlling behavior only; ^b ^Added other measures of control; ^c ^Included relationship inequality; ^d ^Further included demographic and socio-economic characteristics;

Controlling behavior of husband/partner was significantly associated with higher likelihood of sexual violence (RR = 3.65; 95% CI: 2.92 - 4.57) in the unadjusted analysis (Model 1). This association remained highly significant (RR = 4.01; 95% CI: 2.54 - 6.34) after controlling for confounding in Model 4. The likelihood of experiencing sexual violence were higher for women who justify wife beating (RR = 2.20; 95% CI: 1.56 - 3.11), and for women who earned more than their husband/partner (RR = 4.73; 95% CI: 1.54 - 14.51). In contrast, women who had decision-making autonomy (RR = 0.61; 95% CI: 0.42 - 0.90) had a significantly lower likelihood of experiencing sexual violence compared to women who did not have decision-making autonomy (Table 3). All other variables in Tables 2 and 3 had no statistically significant effect on the risks of physical and sexual violence after adjusting for possible confounding.

**Table 3:** Adjusted odds ratios with 95% confidence intervals for controlling behavior and risks of lifetime exposure to sexual violence

	Model 1^a^	Model 2^b^	Model 3^c^	Model 4^d^
	
Characteristics	RR (95% CI)	RR (95% CI)	RR (95% CI)	RR (95% CI)
**Control in relationships**				

*Controlling behavior*				

No	1	1	1	1

Yes	3.65 (2.92 - 4.56)	3.46 (2.70 - 4.43)	3.89 (2.49 - 6.08)	4.01 (2.54 - 6.34)

*Justifying wife beating*				

No		1	1	1

Yes		1.89 (1.57 - 2.27)	2.19 (1.58 - 3.05)	2.20 (1.56 - 3.11)

*Decision-making autonomy*				

No		1	1	1

Yes		0.53 (0.44 - 0.65)	0.55 (0.38 - 0.80)	0.61 (0.42 - 0.90)

**Relationship inequality**				

*Spouses' relative earnings*				

Woman earns same			1	1

Woman earns less			2.27 (0.83 - 6.22)	2.38 (0.87 - 6.56)

Woman earns more			4.70 (1.56 - 14.22)	4.73 (1.54 - 14.51)

Woman's partner does not contribute			1.89 (0.56 - 6.39)	2.03 (0.60 - 6.93)

*Spouses' relative education*				

Woman has same			1	1

Woman has less			0.62 (0.08 - 4.61)	0.64 (0.08 - 4.87)

Woman has more			1.69 (1.22 - 2.35)	1.53 (0.92 - 2.56)

*Spouses' relative age*				

Woman same age			1	1

Woman younger			1.24 (0.30 - 5.18)	1.14 (0.27 - 4.82)

Woman older			0.70 (0.06 - 8.11)	0.69 (0.06 - 7.99)

*Type of union*				

Monogamy			1	1

Polygamy			0.91 (0.64 - 1.29)	1.01 (0.70 - 1.45)

**Demographic & socioeconomic characteristics**				

*Women's age*				

≤ 24				1.30 (0.84 - 2.01)

25 - 34				1.27 (0.89 - 1.83)

≥ 35				1

*Women's education*				

No education				1.07 (0.37 - 3.07)

Primary				1.31 (0.69 - 2.47)

Secondary or higher				1

*Women's occupation*				

Professional/technical/management				1

Clerical/sales/services/skilled manual				0.86 (0.45 - 1.64)

Agricultural self-employed/agricultural employee/household & domestic/unskilled manual				1.04 (0.49 - 2.22)

Not working				0.89 (0.76 - 2.31)

*Partner's education*				

No education				0.60 (0.23 - 1.55)

Primary				0.88 (0.48 - 1.60)

Secondary or higher				1

*Partner's occupation*				

Professional/technical/management				1

Clerical/sales/services/skilled manual				2.22 (0.94 - 5.22)

Agricultural self-employed/agricultural employee/household & domestic/unskilled manual				1.86 (0.76 - 4.59)

*Place of residence*				

Urban				1

Rural				1.39 (0.95 - 2.04)

-2 Log likelihood	5478.06	4707.96	1480.78	1446.31

^a ^Controlling behavior only; ^b ^Added other measures of control; ^c ^Included relationship inequality; ^d ^Further included demographic and socio-economic characteristics;

## Discussion

This present study assessed the effects of controlling behavior by husband/partner control on the risk of intimate partner physical and sexual violence. Findings stress the need to adopt a multidimensional approach to interventions for IPV. Controlling behavior, physical violence and sexual violence against women by husband/partner are largely prevalent in Nigeria, with 13% of the women having reported being exposed to at least one form of physical violence, significantly less than that experienced by women in Turkey (31.3%) [30], older women in the United States (21.9%) [31], and consistent with previous cross-sectional studies in Vietnam (13%) [11]. 63% of the women reported being exposed to at least one form of controlling behavior by their husband/partner, and 3% of the women reported being exposed to at least one form of sexual violence in their lifetime.

Controlling behavior by husband/partner was strongly associated with both physical and sexual IPV, consistent with study findings from other regions [7,11,32], and is a reflection of the increased vulnerability to abuse by women resident in societies that validate male-dominated family structure and social order and encourage men to exercise control over women. This finding is in support of the feminist theory [32], and is also in favor of the hypothesis that controlling behavior is associated with increased likelihood of violence, most likely acting as precursor to violence. However, other factors may be needed to adequately explain this level of violence. Of particular interest also in this study is the variation in the strength of the effects of controlling behavior between physical and sexual violent acts. Husband/partner controlling behavior was associated with three-fold and four-fold higher likelihood of physical and sexual violence, respectively, after adjusting for potential confounders.

Women's justification of traditional societal norms of wife beating by a husband/partner was a strong correlating factor for physical and sexual IPV; consistent with another other study [7]. This is largely regarded as a consequence of societal and cultural factors that permit a man to inflict physical punishment on his wife/partner and the women's acceptance of such violent acts. This level of societal response to partner violence may influence controlling behavior abuse and consequently the likelihood of physical violence [33], and sexual IPV, and is in support of the feminist theory. Decision-making autonomy was associated with reduced likelihood of both physical and sexual IPV, consistent with findings from other studies [34], and contrary to other others [7]. This finding provides support for the *social exchange theory*, and could be based on the premise that a woman's higher status within the household act as protective factors and afford her the ability to resist controls over her life or resist the denial of her rights [35], thereby lessening her vulnerability to abuse if the husband/ partner, as reported in another study [36].

This study also provides evidence of relationship inequalities (women earning more than their spouse) being a strong correlating factor for physical violence, and an even stronger correlating factor for sexual violence, consistent with another study [37], thereby providing support for the *resource perspectives*. Women who neither posses enough economic resources to leave their intimate relationship nor are less able to negotiate change are economically dependent on their husband/partner, making them subject to increased controlling behavior and higher risk of physical violence, as previously shown [38].

Working as agricultural self-employed/agricultural employee/household & domestic/ unskilled manual workers was a strong risk factor for physical violence among the women, consistent with other studies indicating increased risk of physical IPV for women in lower socioeconomic occupations [1]. This may be connected with the fact that within the Nigerian social context, having to combine such physically demanding jobs with domestic responsibilities may not entitle such women the option to redistribution of their domestic responsibilities. The resulting increased tensions within the relationship due to neglected domestic duties, increases the use of controlling behavior and the women's risk of experiencing IPV; similar comments having been raised in another study [39]. Residence in rural areas was a risk factor for physical IPV, consistent with another study [40], and in contrasts to findings others [41]. This may be as a result of rural populations adapting more traditional gender roles than in urban areas, which tends to create environments in which violence in intimate relationships is considered to be more socially acceptable, as recently shown [42]. In addition, poverty tends to be more common in rural areas than in urban or suburban areas; it greatly contributes to family and relationship stress, limits victims' ability to leave abusive intimate partner relationships and increases the vulnerability of the women to physical violence [43]. Other characteristics of rural areas, such as geographic and social isolation, may also increase risks of violence for rural women, and decrease the opportunity for those women who experience violence to seek the resources they need.

### Methodological considerations

Results of the logistic regression analysis were expressed as relative risks (RRs) because relative risks and odds ratios are essentially equivalent under certain circumstances. The *rare disease assumption *i.e. when the probability of an event is low, is one such circumstance. The benchmark commonly used being less than 10% [44,45]. In this study, the lifetime prevalence of controlling behavior by their husband/male partner was high (63%), the lifetime prevalence of physical, and sexual violence were 15% and 3%, respectively.

### Strengths and limitations

The large numbers of respondents, the survey being nationally-representative and enabling the generalization of the results across the country, variables in the DHS surveys being defined similarly across countries making results comparable across countries are major strengths of this study. The limitations of this study include its use of single types of abuse in isolation from the others, which does not control for co-occurrence with other types of violence and its significance for understanding the effects of abuse on victims. The cross-sectional nature of the data makes it difficult to determine causal inference; for instance, it was not possible to determine whether women's justification of wife beating by a husband/partner was influenced by personal experiences of IPV or form traditional norms alone. Finally, the women's report of their husband/partner's use of control tactics may be subject to exaggeration. However, this is considered to be fairly accurate estimates [2].

### Policy implications

This study provided evidence that in countries such as Nigeria in which intimate partner violence is widely accepted in response to women's transgression of traditional gender norms, power within intimate relationships is multidimensional, relative, dependent on the social and cultural contexts, and involves some level of inequity in the distribution of resources. This author believes like others [38], that increasing women's economic resources empower them to bargain for a better situation for them or to leave, therefore, reducing their risk of IPV. Examples of economic interventions, such as microcredit programmer, economic livelihoods, and conditional cash transfers (CCTs), have the potential to enhance decision-making abilities and even reduce IPV [46], through empowerment, as well as address the structural pathways resulting from women's experience of poverty. Another important step toward eliminating this practice is for societies to create social environments that are intolerant towards IPV that would both make it more difficult for perpetrators to persist in their violent behavior and make it less difficult for women to report acts of intimate partner violence [47].

## Conclusions

This study indicated that controlling behavior by husband/partner was strongly associated with physical and sexual violence. It stresses the fact that factors associated with physical and sexual violence are multifaceted, and that the context in which women live predispose them to violence by conferring power upon men to use controlling behavior and acts of physical and sexual violence against women under certain circumstances, such as when women justify wife beating, earn less than their husband/partner, have low status occupations, and reside in rural areas. It also indicates the importance of increasing women's decision-making autonomy as a means of reducing the risk of controlling behavior and acts of physical and sexual violence against women. This study provides evidence for the need for a proactive integrated approach to empower women economically, promote social environments that are intolerant towards controlling behavior and intimate partner violence, thus breaking the norms that sustain women's vulnerability to violence within the society.

## Competing interests

The author declares that they have no competing interests.

## Authors' contributions

DA was responsible for the conceptualization of the study, performed the statistical analysis, and drafting of the manuscript.

## Pre-publication history

The pre-publication history for this paper can be accessed here:

http://www.biomedcentral.com/1471-2458/11/511/prepub
